# Dual Caregivers of Relatives with Dementia in Rural Virginia: The Added Stress of COVID-19

**DOI:** 10.1093/geroni/igaa057.3453

**Published:** 2020-12-16

**Authors:** Emily Hoyt, Jyoti Savla, Karen Roberto, Aubrey L Knight, Rosemary Blieszner, Brandy R McCann

**Affiliations:** 1 Virginia Tech, Blacksburg, Virginia, United States; 2 Carilion Clinic Center for Healthy Aging, Roanoke, Virginia, United States

## Abstract

Family caregivers often find themselves “sandwiched” between caring for an older relative with dementia (PWD) and another person. Serving in a dual caregiving role presents unique challenges and has consequences for caregivers’ physical and mental health. Seven daily diary interviews with 46 dual dementia caregivers assessed their daily stressors and informal and formal supports. Results showed that dementia caregivers who also cared for another older relative reported poorer physical health and used more community-based services to care for the PwD. Conversely, dementia caregivers who also cared for younger relatives reported greater secondary stressors, lower family support, and use of fewer community-based services to care for the PwD. Since the COVID-19 pandemic began, two telephone interviews were conducted with 15 dual dementia caregivers. Caregivers were asked in-depth questions about how the pandemic had impacted their caregiving responsibilities, mental health, and use of community-based services. Guided by stress process and behavior models, a thematic analysis of dual caregivers’ interviews revealed that caregivers had less time for themselves, engaged in self-care activities less often, and felt their social life had suffered. Many of the caregivers reported feeling exhausted, stressed, and had more things to do than they could handle. Of the eight caregivers that used services before COVID-19, six experienced a change in services including loss of services, different workers, or self-selected cancellation of services. Discussion focuses on challenges dual dementia caregivers face and the added stressors they experienced during the COVID-19 pandemic.

